# Endurance and Resistance Training in Radically Treated Respiratory Cancer patients: A Pilot Study

**DOI:** 10.1155/2010/481546

**Published:** 2010-08-05

**Authors:** B. Salhi, I. Demedts, A. Simpelaere, S. Decraene, K. Vermaelen, V. Surmont, J. P. van Meerbeeck, E. Derom

**Affiliations:** Department of Respiratory Medicine, Faculty of Medicine and Health Sciences, Ghent University Hospital, De Pintelaan 185, 7K12IE B-9000 Ghent, Belgium

## Abstract

*Introduction*. Respiratory cancer and its treatment are known to contribute to muscle weakness and functional impairment. *Aim*. To assess the effects of rehabilitation in patients with respiratory cancer. *Methods*. Radically treated respiratory cancer patients were included in a 12-week multidisciplinary rehabilitation program. *Results*. 16 patients (age: 61 ± 7 years; FEV_1_: 57 ± 16% pred.) showed a reduced exercise tolerance (VO_2_max: 56 ± 15% pred.; 6 MWD: 67 ± 11% pred.), muscle force (PImax: 54 ± 22% pred.; QF: 67 ± 16% pred.), and quality of life (CRDQd: 17 ± 5 points; CRDQf: 16 ± 5 points). Exercise tolerance, muscle force, and quality of life improved significantly after rehabilitation. *Conclusion*. Radically treated patients with respiratory cancer have a decreased exercise capacity, muscle force, and quality of life. 12 weeks of rehabilitation leads to a significant improvement in exercise capacity, respiratory muscle force, and quality of life.

## 1. Introduction

Radical treatment for early-stage lung cancer and mesothelioma consists of combinations of surgery, chemotherapy, and radiation therapy [1]. Resection of lung parenchyma reduces pulmonary volumes and exercise tolerance [2]. Chemotherapy (e.g., taxanes, cisplatin) may induce peripheral sensory neuropathy, which can persist for months to years [3]. Radiotherapy, given at doses of 50–66 Gray (Gy), may lead to a functional loss of lung parenchyma, radiation-induced pneumonitis and esophagitis, causing a temporary reduction in food intake. All these treatments are known to contribute to muscle weakness, muscle atrophy, and functional impairment [3–5].

Endurance training has been mostly proposed as a promising strategy in the management of the physical and psychological deterioration in cancer patients. Physical functioning, health-related quality of life (HRQOL), and cancer-related fatigue improve by training in these patients [6, 7].

Two small observational studies suggest that patients with lung cancer may also benefit from posttreatment rehabilitation. Spruit et al. [8] reported that an 8-week inpatient rehabilitation program increased the 6MWD in 10 patients after lung cancer treatment. Another study described that 14 weeks of outpatient rehabilitation improved maximal exercise capacity and the HRQOL in 19 patients [9]. A case-controlled study [10] in 25 lung cancer patients treated by surgery only demonstrated also a significant improvement in 6MWD after a 4-week in-patient rehabilitation program. 

In these three studies, rehabilitation consisted of endurance training. In none of these trials, muscle force was assessed nor was resistance exercise training proposed, although more than 65% of patients reported leg fatigue or a combination of leg fatigue and dyspnea as the major symptom at the end of exercise [9]. 

The present study was designed to investigate (1) whether and to which extent the exercise tolerance, peripheral and respiratory muscle strength, body composition, and dyspnea and fatigue improved by a 12-week outpatient multidisciplinary rehabilitation program, consisting of both resistance and endurance exercise training in patients with radically treated respiratory cancer; (2) whether exercise tolerance after radical treatment was related to peripheral muscle force and mass.

## 2. Materials and Methods

### 2.1. Study Design and Population

The present study was a prospective nonrandomized observational pilot study, conducted in patients with NSCLC or mesothelioma, who were candidate for radical treatment. Radical treatment was defined as surgical resection with or without perioperative chemotherapy, and/or as thoracic radiotherapy with or without chemotherapy. At time point of inclusion, participants had to be radically treated, had to be younger than 75 years, present with a completely normalized blood analysis and exhibit a forced expiratory volume (FEV_1_) <60% of the predicted value, and met at least two of the following criteria: a maximal workload (Wmax) <90 Watt, a 6MWD <70% of the predicted value, <100 points on the chronic respiratory disease questionnaire (CRDQ) or <20 points on the domain dyspnea (CRDQd), and a quadriceps force (QF) <70% of the predicted value or an inspiratory muscle force (PImax) or an expiratory muscle force (PEmax) <70% of the predicted. They all give written informed consent. Patients with severe cardiac, neurological, and orthopaedic comorbidity interfering with exercise training were excluded. The design of the study was approved by the Ethics Committee of the University Hospital Ghent.

### 2.2. Evaluation

Forced expiratory volume in one second (FEV_1_), forced vital capacity (FVC), diffusion capacity in lung (DL,CO) were measured following the ERS guidelines for *pulmonary function testing* (Viasys Sensor medics VMAX, spectra, USA) [11]. Results were expressed as percentages of predicted values [12].

*Body Composition* was assessed using a single frequency (50 kHz) bioelectrical impedance device (Bodystat 1500 Medical, Isle of Man, LTD). The electrical current was applied to the skin through four adhesives electrodes attached to the right dorsal side of wrist and foot, while the patient was in supine position. Body mass index (BMI) was defined from height and weight. The Fat Free Mass index (FFMI) was calculated as the ratio of FFM (Fat Free Mass) to height in squared meters. Nutritional depletion was defined as BMI ≤20 kg/m^2^ and/or FFMI ≤15 (females) or ≤16 (males) kg/m^2^[13].

*Maximal Exercise Capacity (Wmax)* was assessed by an incremental symptom-limited exercise test (Jaeger cycle-ergo meter). Heart rate, blood pressure, and oxygen saturation were continuously monitored during the procedure. After 3 minutes of unloaded pedalling, workload was increased by 10 Watt/min until exhaustion. Oxygen consumption, carbon dioxide output, and ventilation were measured breath by breath. Maximal oxygen consumption (VO_2_max) was compared with normal values [14]. Exercise limitation was determined according to the ATS/ACCP guidelines for cardiopulmonary exercise testing [15].

*6 Minute Walking Distance (6MWD)* was measured according to the ATS-guidelines [16]. Oxygen saturation was measured continuously. The best out of 3 attempts was retained and expressed as a percentage of the predicted value [17].

*Inspiratory and Expiratory Muscle Strength (PImax and PEmax) * was determined according to the method of Black and Hyatt [18]. Expiratory muscle strength was measured starting from TLC and inspiratory muscle strength starting from RV. Tests were repeated until the variability among three attempts was less than 5%. The highest value was expressed as a percentage of the predicted value [19].

*Hand-Grip Force (HGF)* was measured using a hydraulic hand dynamometer. (Jamar Preston, Jackson, MI, USA). Peak HGF was assessed at the dominant side with the elbow at 90° flexion, with the undergroup and wrist in neutral position. Three attempts were performed and the highest value was taken for analysis and expressed as a percentage of the predicted value [20].

*Isometric Quadriceps Force (QF)* was measured using an isometric handheld dynamometer (Microfet; Biometrics, Almere, the Netherlands) attached on a knee pendicular bank. Extension peak torque was evaluated at 60° of knee flexion. Patients were asked to perform a 5 seconds isometric contraction. The best out of three attempts was retained and expressed as a percentage of the predicted value [21].

*Dyspnea and Fatigue* were assessed using the domains “dyspnea” and “fatigue” of the validated Dutch translation of the CRDQ (Chronic Respiratory Disease Questionnaire) [22].

### 2.3. Multidisciplinary Rehabilitation Program

At intake, every patient was examined by a pulmonologist, a physiotherapist, a social worker, a nutritionist, a psychologist, and an occupational therapist. All programs were individually tailored. Each rehabilitation session consisted of 60 minutes of reconditioning and 30 minutes of occupational therapy, nutritional support, or psychosocial support depending on the personal needs of every patient.

Exercise training included bicycle training, treadmill walking, stair climbing, and peripheral muscle training on Technogym equipment (leg press, leg extension, abduction and adduction, the vertical row, vertical tracker, and chest press). Resistance training for peripheral muscles of the upper and lower extremity started with 3 series of 10 repetitions at 50% of the 1 RM (one Repetition Maximum, weight that could be lifted once). Subsequently, the resistance was progressively increased to 80% of the 1 RM to reach a Borg score of 4–6 for dyspnea or fatigue [23]. New 1 RM measurements were performed after 6 weeks. Endurance training consisted of walking on a treadmill and cycling on a bicycle ergometer. Treadmill training intensity started at 70% of the average speed of the 6MWD for 6 minutes in the first week and was subsequently enhanced by increasing duration, speed, and eventually slope. Bicycle training intensity started at 70% of the average workload of the maximal incremental test for 10 minutes and the duration and load were progressively enhanced until the 12th week. During each session, workload and maximal walking speed were adapted to reach a Borg score between 4 and 6 for dyspnea and fatigue [23]. 

We systematically prescribed nutritional support by dietary advice and oral nutritional supplements in accordance with criteria proposed in other studies: BMI <18.5 kg/m^2^; unintentional weight loss >10% within the last 3–6 months; or BMI <20 kg/m^2^ and unintentional weight loss >5% within the last 3–6 months [24].

### 2.4. Data Analysis

The change in 6MWD after 12 weeks was used as primary outcome. Changes in QF, PImax, PEmax, HGF, VO_2_max, Wmax, FFM, FFMI, CRDQd, and CRDQf were secondary outcomes. The analysis was performed using nonparametric statistics of the Statistical Package for the Social Sciences (SPSS 15.0). The Wilcoxon test was used to see a difference between the variables at intake and after 12 weeks. A significant level of *P* < 0.05 was used throughout the data analysis. Clinically relevant improvements in 6MWD, Wmax, and CRDQd were defined as a progression exceeding 54 m, 10 Watt, and 2.5 points, respectively [25–27]. Spearman analysis was used to detect correlations between the exercise capacity and the muscle force and mass. All values are expressed as means and standard deviations (SD).

## 3. Results

All 16 patients, included in the study (Male: 13; age: 61 ± 7 years; FEV_1_: 57 ± 15% pred.; FVC: 66 ± 28% pred.; DL, CO: 43 ± 15% pred.) completed the entire 12 week training program. They were all considered to be radically treated at intake. Ten out of 16 patients were diagnosed with NSCLC, 4 patients were diagnosed with mesothelioma and two with a carcinoid tumour. Nine patients underwent a pneumonectomy and five a lobectomy. Eleven of them were treated with either adjuvant chemotherapy or radiotherapy (Table 1). Intake for rehabilitation took place 18 ± 4 weeks after completion of radical treatment. Adverse effects were not reported.

Maximal exercise testing at baseline revealed that eight patients reached their maximal predicted ventilation and one patient his maximal predicted heart rate. The remaining seven patients performed also a maximal exercise test but neither reached their cardiac nor their ventilatory limitation and were thus assumed to be limited by peripheral muscle weakness. 

**Figure 1:**
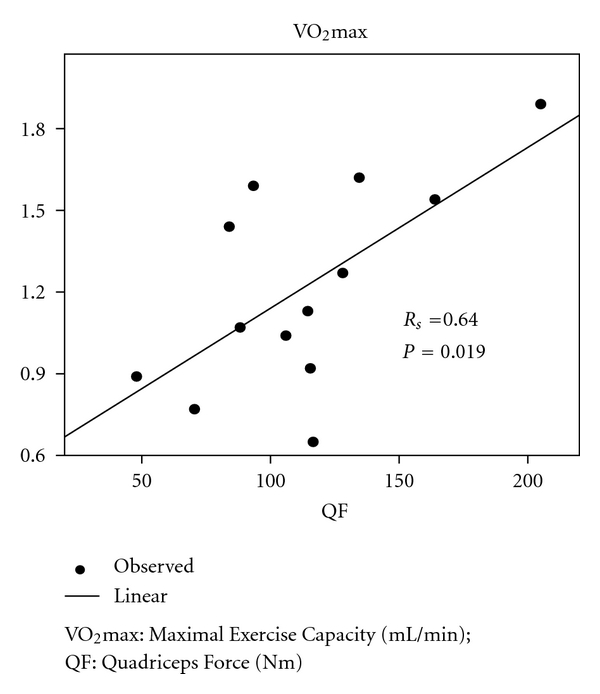
Correlation between maximal exercise capacity and muscle force.

Exercise tolerance and muscle force were reduced to approximately 60% of the predicted value, whereas dyspnea and fatigue were increased at baseline (Table 2). After 12 weeks of rehabilitation, Wmax increased significantly from 83 ± 40 Watt to 103 ± 55 Watt and VO_2_max from 1129 ± 393 mL/min to 1345 ± 527 mL/min (*P* < 0.05). 6MWD improved significantly from 470 ± 88 m to 533 ± 85 m (*P* < 0.05) (Table 2). Peripheral and respiratory muscle force also improved, but only the increase in PImax reached statistical significance (Table 2). There was a statistically significant correlation between QF and VO_2_max at inclusion (*R*_*S*_ = 0.64; *P* = 0.019) (Figure 1). 

HRQOL improved at the end of the program significantly; CRDQd increased from 17 ± 5 points to 24 ± 6 points and CRDQf from 16 ± 5 points to 18 ± 5 points (*P* < 0.05) (Table 2).

After 12 weeks of rehabilitation, improvement exceeding the minimal clinically significant difference was reached for 6MWD (59 ± 52 m), Wmax (22 ± 25 Watt), and CRDQd (7 ± 5 points). 

**Figure 2:**
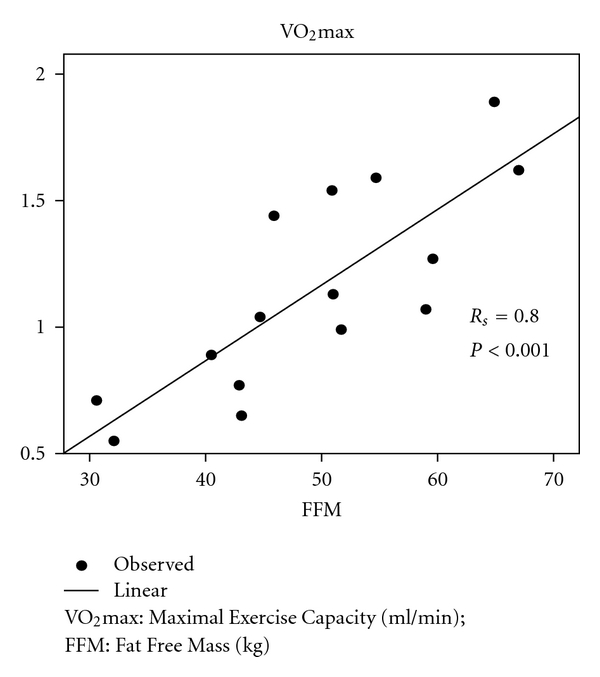
Correlation between maximal exercise capacity and muscle mass.

25% of the patients exhibited a BMI ≤20 kg/m^2^ at inclusion (Table 2). At the end of the pulmonary rehabilitation program, only 10% of patients had a BMI ≤20 kg/m^2^. FFMI was reduced in 47% of patients at intake and in 43% of the patients at the end of the program. There was a statistically significant correlation between the FFM and VO_2_max at baseline (*R*_*S*_ = 0.80,  *P* < 0.001) (Figure 2), also between FFM and Wmax, and 6MWD and QF (*R*_*S*_ = 0.86, *P* < 0.001; *R*_*S*_ = 0.56, *P* = 0.03; *R*_*S*_ = 0.64, *P* = 0.03).

## 4. Discussion

This observational pilot study shows that radically treated patients with respiratory cancer suffer from impaired exercise capacity, increased levels of dyspnea and fatigue and reduced peripheral and respiratory muscle force. Admittedly, our study was neither randomized, nor controlled. Although spontaneous recovery could have explained our results, there is a lot of evidence that radical treatment in lung cancer patients has downbeat effects on pulmonary function, exercise capacity, and quality of life, which last for at least 6 months [2, 28]. In some studies, recovery never occurred or decreases in pulmonary function and 6MWD over time have been reported [10]. 

The present study also demonstrates that radically treated patients for lung cancer experience beneficial effects from a multidisciplinary pulmonary rehabilitation program, provided that they present with impaired exercise capacity, fatigue, and dyspnea at inclusion. Interestingly, none of the previous studies used such strict inclusion criteria as we did. Actually, this study was conducted because over the last years patients were increasingly referred by their thoracic oncologist. We seized that opportunity to assess the effect of rehabilitation. 

VO_2_max after radical treatment was reduced to 56% pred. and 6MWD to 68% pred. in our patients. This is in line with data reported previously in lung cancer patients [8, 9]. It is unclear to which extent the underlying malignancy or its treatment might have contributed to this reduction in exercise tolerance. Data by Nezu et al., who observed that maximal exercise capacity after lung resection did not reach preoperative values after more than 6 months, tend to suggest that the factor “treatment” could have played a prominent role in this regard [2]. However, the potential contribution of the cardiovascular, ventilatory, or musculoskeletal system remains to be systematically investigated in these patients. 

Although studies on the incidence of peripheral and respiratory muscle weakness after radical treatment for lung cancer or mesothelioma have not been conducted so far, we found out that respiratory cancer patients exhibited a substantial decrease in respiratory and peripheral muscle force. Indeed in 64% of our patients an abnormally low QF and in 50% of patients a decreased FFM were observed. These observations, together with the presence of peripheral limitation at maximal exercise in half of the patients as well as the significant correlation between VO_2_max and FFM, support the hypothesis that muscle weakness might be a critically important determinant of exercise limitations in patients radically treated for respiratory cancer. 

Our rehabilitation program consisted of both resistance and endurance training. Numerous studies have documented the physiological benefits of resistance training in terms of increased muscle mass and strength in healthy subjects. Resistance training increases the cross-sectional area of the muscle (hypertrophy), allowing the muscle to generate more force. Peripheral and respiratory muscle strength increased in the present study, but only PImax reached statistical significance. Possibly a larger number of sessions or a more intensive program could have been requested to transfer the training stimuli into real gains in muscle force as has been reported after rehabilitation of patients with COPD and restrictive lung diseases [29, 30]. Another possible explanation could be that the sample size was too small to obtain statistically significant improvements for the peripheral muscles. The increase in Pimax might have been attributed to the fact that in our series, 60% of patients had respiratory muscle weakness and were asked to train their inspiratory muscles every day starting at 30% PImax for 20 minutes. Such improvements have been reported in many trials in which the effect of IMT has been assessed [23].

It has been suggested that nutritional support in patients with cancer may improve nutritional status, quality of life, functional capacity, and lean mass [31]. Our data tend to support this issue, we systematically prescribed nutritional support to nutrionally depleted patients, since 70% of patients received nutritional support, which resulted in as statistically significant improvement in FFMI and FFM by 2 kg. It remains unclear whether an increase in muscle force and/or mass might improves the prognosis of these patients, although loss of skeletal muscle mass is considered to be a bad prognostic factor. The present findings provide a rationale to investigate the effect of rehabilitation on survival in radically treated lung cancer patients. 

Although the improvement in QF was not statistically significant, exercise capacity improved in our patients after 12 weeks. This is in line with the results of an observational study of inpatient rehabilitation, showing a substantial improvement of the 6MWD after 8 weeks [8]. A second study, consisting of an inpatient rehabilitation program of 4 weeks resulted in an improvement of the Borg scale on exertion, the 6MWD, and the hemoglobin saturation during the walking test in 25 patients, compared to 186 patients who had refused to participate in the training group and served as controls [10]. A third study by Jones et al. also showed a significant improvement of the exercise tolerance and HRQOL after 14 weeks of outpatient rehabilitation in radically treated patients with NSCLC. Our study, which lasted for 12 weeks, demonstrated much larger improvements in exercise tolerance in comparison with the results of Jones et al. (mean difference VO_2_max % pred.: 22% versus 4%; mean difference Wmax: 22 Watt versus 9 Watt) [9]. Perhaps the integration of resistance training in our program might have caused the present results.

Increases in HRQOL are as important as increases in physiological outcomes in rehabilitation studies. The CRDQ has been specially designed for the assessment of HRQOL in COPD patients. Some of the domains have been used in patients with restrictive lung disorders [30, 32]. The current study demonstrated that fatigue and dyspnea decreased significantly after 12 weeks of rehabilitation. These findings are highly relevant, since fatigue remains the most frequently reported symptom in cancer patients and is an important determinant of a low HRQOL [7]. Studies in patients with breast cancer indicate that endurance training is the modality of choice to decrease cancer-related fatigue [6, 7, 33]. In the present study, a combination of resistance and endurance training was prescribed, in order to treat both the muscle weakness and the cancer-related fatigue.

## 5. Conclusions

Patients with radically treated respiratory cancer suffer from a quite substantial decrease in exercise capacity and muscle force and an increased level of dyspnea and fatigue. Significant improvements in exercise capacity, respiratory muscle force, dyspnea, and fatigue were obtained after a 12-week multidisciplinary pulmonary rehabilitation program. The results of the present observational study, consisting of a small number of patients, require additional confirmation in larger prospective randomized clinical trials, which may establish the relevance of rehabilitation in the management of patients with respiratory cancer. Such studies may also solve other issues, such as the characteristics of the patients with respiratory cancer who may benefit most from rehabilitation after radical treatment, the duration of a pulmonary rehabilitation program, the optimal training modality for these patients, and the importance of nutritional support remain to be solved.

## Figures and Tables

**Table 1:** Patient characteristics: Diagnose, Treatment, and Stage.

Patients	Age (years)	Gender	Tumour type	Treatment	Post operative stage	Post operative Hb (g/dL)
1	67	F	Atypical carcinoid tumour	Pneumonectomy + Chemotherapy	III A	9.7
2	51	M	NSCLC	Chemotherapy + Radiotherapy	III B	13.1
3	53	M	NSCLC	Pneumonectomy	II A	13.7
4	54	M	Pleural mesothelioma	Pneumonectomy + Chemotherapy + Radiotherapy	III	12.8
5	66	M	NSCLC	Pneumonectomy + Chemotherapy	III A	11.7
6	79	M	NSCLC	Pneumonectomy	III B	11.2
7	66	M	Pleural mesothelioma	Pneumonectomy + Chemotherapy + Radiotherapy	III	12.9
8	66	F	NSCLC	Lobectomy	I B	13.8
9	58	M	NSCLC	Lobectomy + Chemotherapy	III B	14.6
10	70	M	NSCLC	Lobectomy + Radiotherapy	IB	10.8
11	61	M	Pleural mesothelioma	Pneumonectomy +Chemotherapy + Radiotherapy	III	10
12	55	M	NSCLC	Lobectomy + Radiotherapy	IIB	12.4
13	62	M	NSCLC	Pneumonectomy	IIIB	15.5
14	51	M	Pleural mesothelioma	Pneumonectomy +Chemotherapy + Radiotherapy	III	14.6
15	64	F	NSCLC	Radiotherapy	IA	15.8
16	64	M	Typical carcinoid tumour	Bilobectomie	IA	13

NSCLC: Non Small Cell Lung Cancer.

**Table 2:** The effects of a 12-week multidisciplinary pulmonary rehabilitation program.

	At baseline (*n* = 16)	After 12 weeks (*n* = 16)	*P* value
FEV_1_ (L)	1.8 ± 0.7	1.7 ± 0.6	*P* = .629
BMI (kg/m^2^)	23 ± 5	24 ± 5	*P* = 0.060
VO_2_max (% pred.)	56 ± 15	66 ± 15	*P* = 0.022
6 MWD (% pred.)	68 ± 11	78 ± 9	*P* = 0.003
Quadriceps force (% pred.)	67 ± 17	78 ± 31	*P* = 0.505
Handgrip force (% pred.)	71 ± 31	74 ± 33	*P* = 0.209
PImax (% pred.)	54 ± 23	62 ± 19	*P* = 0.041
FFM (kg)	49 ± 11	51 ± 13	*P* = 0.025
FFMI (kg/m^2^)	16 ± 3.0	17 ± 3.0	*P* = 0.024
CRDQd (points)	17 ± 5	24 ± 6	*P* = 0.002
CRDQf (points)	16 ± 5	18 ± 5	*P* = 0.027

FEV_1_: Forced Expiratory Volume in 1 sec; BMI: Body Mass Index; VO_2_max: Maximal Oxygen Consumption; 6MWD: 6-Minute Walking Distance; PImax: Maximal Inspiratory Pressure; FFM: Fat Free Mass; FFMI: Fat-Free Mass Index; CRDQd: Chronic Respiratory Disease Questionnaire dyspnea; CRDQf: Chronic Respiratory Disease Questionnaire fatigue.
